# Supplementary material for: HIVprotI: an integrated web based platform for prediction and design of HIV proteins inhibitors
Source: J Cheminform. 2018 Mar 9;10:12. doi: 10.1186/s13321-018-0266-y (PMC5845081; doi:10.1186/s13321-018-0266-y)
Supplement: Supplementary file 2 — Additional file 2. Source code of HIVProtI web server. [file 13321_2018_266_MOESM2_ESM.zip › HIVprotI_Source-code/hivprotI/weka-3-6-6/doc/weka/associations/tertius/Body.html]

Body


---


|  |  |  |
| --- | --- | --- |
| **Overview** \| **Package** \| **Class** \| **Tree** \| **Deprecated** \| **Index** \| **Help** \| **Weka's home** | |  |
| **PREV CLASS**   **NEXT CLASS** | **FRAMES**    **NO FRAMES**     **All Classes** |  |
| SUMMARY: NESTED \| FIELD \| CONSTR \| METHOD | DETAIL: FIELD \| CONSTR \| METHOD |  |


---


## weka.associations.tertius Class Body

```
java.lang.Object
  weka.associations.tertius.LiteralSet
      weka.associations.tertius.Body
```

**All Implemented Interfaces:**: java.io.Serializable, java.lang.Cloneable, RevisionHandler

---

``` public class Body extends LiteralSet ```

Class representing the body of a rule.

**Version:**
:   $Revision: 1.6 $

**Author:**
:   Amelie Deltour

**See Also:**: Serialized Form

---

**Constructor Summary**

`Body()`
  
          Constructor without storing the counter-instances.

`Body(Instances instances)`
  
          Constructor storing the counter-instances.


| **Method Summary** | |
| --- | --- |
| `boolean` | `canKeep(Instance instance, Literal newLit)`             Test if an instance can be kept as a counter-instance, if a new literal is added to this body. |
| `java.lang.String` | `getRevision()`             Returns the revision string. |
| `boolean` | `isIncludedIn(Rule otherRule)`             Test if this Body is included in a rule. |
| `java.lang.String` | `toString()`             Gives a String representation of this set of literals as a conjunction. |

**Methods inherited from class weka.associations.tertius.LiteralSet**

`addElement, clone, contains, counterInstance, counterInstance, enumerateLiterals, getCounterInstancesFrequency, getCounterInstancesNumber, getLastLiteral, getType, hasMaxCounterInstances, isEmpty, negationIncludedIn, numLiterals, overFrequencyThreshold, upDate`

**Methods inherited from class java.lang.Object**

`equals, getClass, hashCode, notify, notifyAll, wait, wait, wait`

**Constructor Detail**

### Body

```
public Body()
```

:   Constructor without storing the counter-instances.

---


### Body

```
public Body(Instances instances)
```

:   Constructor storing the counter-instances.

    **Parameters:**: `instances` - The dataset.


**Method Detail**

### canKeep

```
public boolean canKeep(Instance instance,
                       Literal newLit)
```

:   Test if an instance can be kept as a counter-instance,
    if a new literal is added to this body.

    :   **Specified by:**: `canKeep` in class `LiteralSet`
    :   **Parameters:**: `instance` - The instance to test.: `newLit` - The new literal. **Returns:**: True if the instance is still a counter-instance (if the new literal satisfies the instance).

---


### isIncludedIn

```
public boolean isIncludedIn(Rule otherRule)
```

:   Test if this Body is included in a rule.
    It is the literals of this Body are contained in the body of the other rule,
    or if their negation is included in the head of the other rule.

    :   **Specified by:**: `isIncludedIn` in class `LiteralSet`
    :   **Parameters:**: `otherRule` - The rule to test. **Returns:**: True if this set of literals is included in the rule.

---


### toString

```
public java.lang.String toString()
```

:   Gives a String representation of this set of literals as a conjunction.

    :   **Specified by:**: `toString` in class `LiteralSet`

---


### getRevision

```
public java.lang.String getRevision()
```

:   Returns the revision string.

    :   **Returns:**: the revision


---


|  |  |  |
| --- | --- | --- |
| **Overview** \| **Package** \| **Class** \| **Tree** \| **Deprecated** \| **Index** \| **Help** \| **Weka's home** | |  |
| **PREV CLASS**   **NEXT CLASS** | **FRAMES**    **NO FRAMES**     **All Classes** |  |
| SUMMARY: NESTED \| FIELD \| CONSTR \| METHOD | DETAIL: FIELD \| CONSTR \| METHOD |  |


---
